# Geripausal Women—A New Challenge for Urogynecology in Upcoming Years

**DOI:** 10.3390/jcm15020530

**Published:** 2026-01-09

**Authors:** Aleksandra Kołodyńska, Aleksandra Kamińska, Aleksandra Strużyk, Ewa Rechberger-Królikowska, Magdalena Ufniarz, Tomasz Rechberger

**Affiliations:** 2nd Department of Gynecology, Medical University of Lublin, 20-059 Lublin, Poland; aleksandra.kolodynska@umlub.edu.pl (A.K.); aleksandra.kaminska@umlub.edu.pl (A.K.); aleksandra.struzyk@usk4.lublin.pl (A.S.); ewarechberger92@gmail.com (E.R.-K.); magdalena.ufniarz@umlub.edu.pl (M.U.)

**Keywords:** geripause, elderly women, urogynecology, pelvic floor disorders, pelvic organ prolapse, stress urinary incontinence, overactive bladder

## Abstract

**Background/Objectives:** The growing population of women aged ≥ 80 years poses a new challenge for urogynecology. Advanced age, comorbidities, and polypharmacy raise concerns regarding the safety of procedures in the management of pelvic floor disorders (PFDs) such as pelvic organ prolapse (POP), stress urinary incontinence (SUI), and overactive bladder (OAB). Individualized, frailty-based assessment is essential in this group. The aim of the study was to evaluate the safety profile of urogynecological surgical procedures among women aged ≥ 80 years at a single tertiary center. **Methods:** In a retrospective observational single-center study, we analyzed the medical documentation of 774 hospitalizations of women aged ≥ 80 years admitted between 2014 and 2023. The analysis included indications, comorbidities, treatment types, anesthesia, and complications. Comorbidity and surgical risk were evaluated using the Charlson Comorbidity Index (CCI) and Clavien–Dindo classification. **Results:** A total of 720 admissions with complete medical records were analyzed, of which 65% were for urogynecological conditions. In this group, the mean age was 83.0 years and mean BMI was 27.2 kg/m^2^. Most patients (92.9%) had comorbidities, mainly hypertension (84.2%) and diabetes (21.1%). POP was the leading indication (52%), followed by SUI (35%) and OAB (27%). Surgical management was performed in 95% of POP cases, predominantly via vaginal native tissue repair (80%), especially LeFort colpocleisis (20%). The transobturator sling (TOT) was the most frequent SUI surgery. Intraoperative complications occurred in 1.5% of cases and postoperative ones were mainly minor (Clavien–Dindo I–II). No procedure-related deaths were recorded. **Conclusions:** In this cohort, surgical treatment of urogynecological problems in women ≥80 years was associated with a low rate of major complications, suggesting that it can be safely offered to elderly patients. Careful preoperative assessment based on frailty and comorbidity rather than chronological age remains essential.

## 1. Introduction

The topic of geriatric urogynecology is currently receiving much attention. This is mainly due to current demographical trends which clearly indicate that, in the mid-2030s, the global population of people aged 80 and above is expected to reach 265 million, surpassing the number of infants [1]. This is of course a consequence of the extension of female life expectancy, and consequently, the demand for urogynecological care is expected to grow. Therefore, urogynecologists all over the world should expect a growing demand for counseling and effective treatment for patients with pelvic floor defects, regardless of the fact that elderly women are more likely to suffer from other comorbidities which may aggravate preoperative and postoperative lower urinary tract symptoms (LUTSs) as well. Some prognoses predicted more than a 50% increase for urogynecological procedures in upcoming years [2]. Recently, a sizeable debate began concerning the standardization of surgical techniques for the surgical treatment of pelvic floor dysfunctions, especially due to the statements of the Food and Drug Administration (FDA) regarding the usage of artificial prostheses in urogynecology. However, we should appreciate that female geripause itself could be an intrinsic, independent biological factor which can influence both the surgery and its final outcome as well [3]. Surgeons should remember that advanced age itself, regardless of accompanying comorbidities, will increase the risk of postoperative complications when compared to younger counterparts undergoing the same surgical procedures [4]. On the other hand, in the near future, there will definitely be a marked increase in demand for operative intervention, even assuming an increase in perioperative and postoperative morbidity and mortality when compared to the younger population. Chronological age alone, however, should not be used to guide treatment decisions, as it does not take frailty into account [5]. There is a large quantity of evidence clearly showing that frailty scores perform better than chronological age alone at predicting the prognosis after surgery, especially in oncological patients [6]. Any type of surgical insult, regardless of its invasiveness, is a form of acute stress that requires an increased physiological response from elderly patients who are at obvious risk of postoperative complications. Surgeons often choose to simplify surgery in the hope of reducing complications and improving survival, but this decision is often based on chronological age alone. On the other hand, frailty indices such as the modified Charlson Comorbidity Index (CCI), which has a strong predictive accuracy for mortality, could be useful for guiding treatment decisions in such cases [7].

The aim of this retrospective observational study was to analyze the clinical indications for urogynecological admission of octogenarian patients (over 80 years old) and to evaluate the perioperative safety of surgical procedures performed in this group over a 10-year period at a single tertiary center.

## 2. Materials and Methods

This was a retrospective observational study conducted in a tertiary gynecology department. All admissions of patients aged 80 and older were reviewed between January 2014 and December 2023.

**Figure 1:**
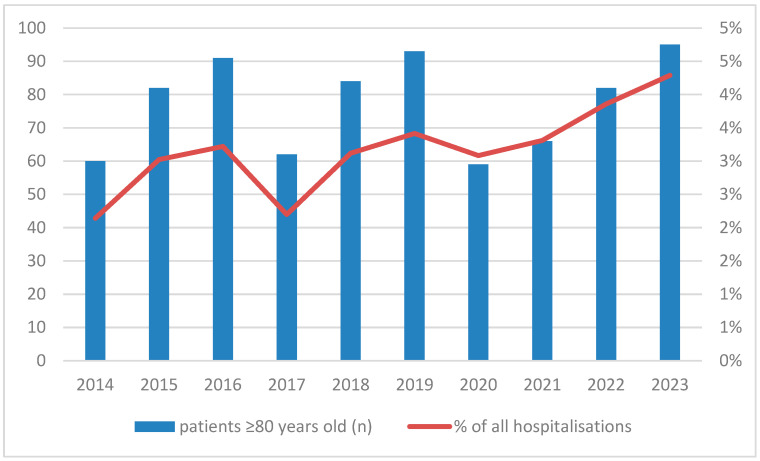
Increasing trends of octogenarian (>80 years old) and nonagenarian (>90 years old) admissions.

In the analyzed period, the growing trend of elderly patient (≥80 years old) hospitalizations percentage among all was observed [Figure 1].

The inclusion criteria included patients ≥ 80 years at the day of admission, urogynecological condition as an indication for hospitalization, and complete medical documentation available. A total of 774 cases were screened; 720 included in the final analysis after excluding incomplete documentation. Of these, 65% (*n* = 469) met all the inclusion criteria.

The extracted variables included demographic characteristics of the group (age, BMI, parity), comorbidities and Charlson Comorbidity Index (CCI), diagnosis, type of intervention, anesthesia type (if applicable), and complications.

The primary outcome of the study was perioperative safety, assessed through intraoperative complications, postoperative complications within hospitalization, and severity of complications graded using the Clavien–Dindo classification. No long-term outcomes were assessed in the study.

Data were summarized using descriptive statistics (means, ranges, frequencies). Analyses were performed using STATISTICA 13 software from StatSoft Polska Sp. z o.o., (Cracow, Poland) standard statistical software.

The research was approved by the local ethics committee (KE-0254/75).

## 3. Results

### 3.1. Characteristics of the Group

The analyzed group included 469 patients aged ≥ 80 years. The mean age was 83.02 (xmin = 80; xmax = 93).

The BMI ranged between 14.9 and 42.2 kg/m^2^ (mean 27.2 kg/m^2^). The parity is described in Table 1.

Most of the patients (92.96%) suffered from various comorbidities. The most common comorbidities are described in Table 2. The polypharmacy, described as chronic use of ≥5 medications or supplements, was found in 47.76% patients.

According to the comorbidities and medical history of the patients, we also assessed the predicted 10-year survival by Charlson Comorbidity Index (CCI) [8]. Most of the patients received 4 points, which is associated with a 53% chance of 10-years survival (see Table 3). It is crucial to underline that the 4 points are given solely due to the age of ≥80 years old.

The largest group of patients presented pelvic organ prolapse (*n* = 242, 52%). The Second most common urogynecological problem was stress urinary incontinence (*n* = 162, 35%). One in four patients suffered from overactive bladder symptoms (*n* = 129, 27%). The least common condition was vaginal fistulas (*n* = 19, 4%) or others (e.g., urine retention, infection, exposition of the vaginal mesh after previous surgery; *n* = 11, 2%).

### 3.2. Pelvic Organ Prolapse (POP)

As the main reason for hospitalization, pelvic organ prolapse was identified among 242 patients. The POP-Q scale assessment is described in Table 4.

More than 95% of the patients (*n* = 231) were scheduled for the surgical treatment, 3% of the patients (*n* = 8) were temporarily or permanently disqualified from the surgical treatment due to comorbidities, and three patients (1.2%) did not sign the informed consent for the proposed therapy and were discharged.

The commonly used techniques were vaginal native tissue repair (VNTR) (*n* = 186, 80%), including both reconstruction (vaginal colporraphy and kolpoperineomyoplasty, transvaginal hysterectomy (TVH) with apical suspension and Manchester repair) and obliterative techniques (LeFort colpocleisis). The high prevalence of the LeFort technique (*n* = 50) seems explainable due to its high efficacy and the percentage of patients who no longer have vaginal sexual intercourse.

Transvaginal mesh insertion (TVM) was performed in 43 cases (19%) and sacrocolpopexy in 2 cases (1%).

**Figure 2:**
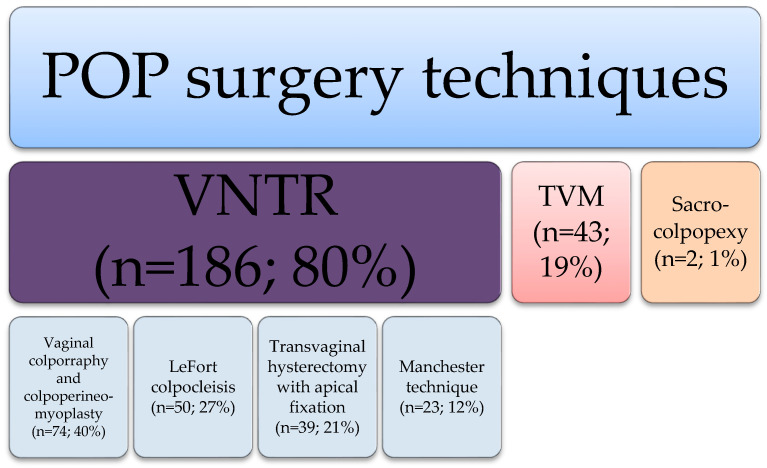
Pelvic organ prolapse repair techniques used in analyzed group.

More detailed data are presented in Figure 2.

About 75% of the patients (*n* = 175; 75.76%) underwent the surgery under general anesthesia, more than 20% under subarachnoid anesthesia (*n* = 54; 23.38%), and only in two cases was the surgery performed under local anesthesia (*n* = 2; 0.86%).

Intraoperative complications appeared in seven cases: six urinary bladder disruptions (four—TVM insertion, one—anterior kolporraphy, one—Manchester technique) and one rectum perforation (TVM insertion). All of the complications were diagnosed and repaired during the initial surgery with no long-term adverse effects.

The postoperative complications were assessed on the Clavien–Dindo scale, which is based on the type of therapy needed to treat it [9]. There were no complications that caused the permanent disability or death of any patient; more detailed data are presented in Table 5.

### 3.3. Stress Urinary Incontinence (SUI)

In the analyzed group, 163 patients reported the symptoms of stress urinary incontinence, in 34 cases SUI was concomitant with the POP, and in 35 cases patients presented mixed urinary incontinence with SUI predominance.

The surgical treatment was performed in 123 patients, 12 patients had the bulking agents injection, and 2 patients underwent platelets-rich plasma injection in the periurethral area.

**Figure 3:**
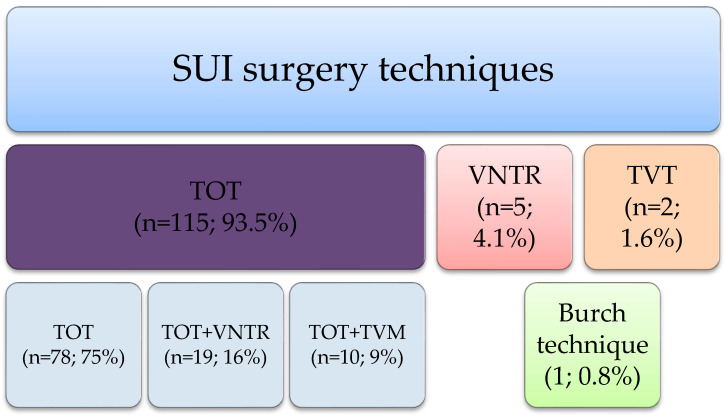
Stress urinary incontinence surgery techniques used in the analyzed group.

The most common technique performed for SUI was transobturator miduretheral sling (TOT); detailed information about the techniques used are presented in Figure 3.

Most of the surgeries were performed under general anesthesia (*n* = 111; 98.2%), 11 under subarachnoid anesthesia (9%), and 1 under local anesthesia.

There was only one case of intraoperative complication—urinary bladder perforation, which was simultaneously repaired. There were no serious postoperative complications; the detailed characteristics according to the Clavien–Dindo classification are given in Table 6.

### 3.4. Overactive Bladder Syndrome (OAB)

There were 129 patients who reported urge urinary incontinence. The majority of patients with OAB itself or MUI with dominantly OAB symptoms were qualified for the cystoscopy with botulin toxin urinary bladder injection (*n* = 89; 69%). Two patients had only the cystoscopy performed—due to the oncologically alarming changes in the bladder’s mucosa, the patients were disqualified from the procedure. Three patients were disqualified due to urinary tract infection. The MUIs with the SUI predominance (*n* = 35) were scheduled for the appropriate treatment (*n* = 33 TOT; *n* = 1 bulking agent injection; *n* = 1 platelets-rich plasma injection).

The botulin toxin injections were performed under local anesthesia and had no intraoperative complications.

### 3.5. Vaginal Fistulas

There were 19 patients hospitalized due to vaginal fistulas: 17 vesicovaginal fistulas (VVF), 1 urethrovaginal fistula, and 1 rectovaginal fistula. In the VVF group, eight patients had the Latzko technique vaginal fistula repair performed, and five patients had the PRP injection in case of surgical treatment failure. Four patients were disqualified from the surgical treatment due to advanced infection of the urinary tract or the vagina.

The patient with urethrovaginal fistula had a midurethral sling removal under general anesthesia.

The patient with rectovaginal fistula did not sign the informed consent for the surgical treatment and was discharged.

The Latzko surgeries were performed mostly under general anesthesia (*n* = 6), and two surgeries were performed under subarachnoid anesthesia. There were no intraoperative complications after these procedures.

There were only Grade 1 complications according to the Clavien–Dindo scale—two patients required additional antiemetics in the early postoperative period.

### 3.6. General Perioperative Safety Outcomes

Intraoperative complications occurred in 1.5% of all procedures. The common complication was bladder injury, which was repaired immediately every time during the primary operation.

According to the Clavien–Dindo classification, the most frequently observed Grade I complications required only minor pharmacological intervention. Grade II complications primarily consisted of urinary retention necessitating catheterization or additional pharmacological management. Grade III events were uncommon and included cases requiring hematoma evacuation or tape removal. No Grade IV–V complications were identified.

## 4. Discussion

It is estimated that 15–30% of community-residing elderly women and more than half of nursing home residents have UI and 23% women with UI used pads or diapers regularly [10,11]. The growing number of octogenarians with PFD emphasizes the need for seeking safe and successful therapeutic resolutions.

Intraurethral pressure (IUP) normally is provided by smooth muscles, connective tissue, and submucosal vascular plexus. Aging can impair IUP because of degenerative changes in elastic connective tissue, decreases in blood flow and vascular pulsation, and reductions in the number and density of urethral smooth-muscle fibers [12].

Decreased bladder volume, more frequent involuntary bladder contractions, and lowered resistance of the urethra predispose towards urinary incontinence (UI) and urinary tract infection (UTI) among elderly patients [10].

Age-related changes such as muscle atrophy (sarcopenia), decreased motility and sensory perception, and cerebral insults also contribute. Smoking, parity, hysterectomy, and other gynecological operations may increase the risk for UI. The postmenopausal estrogen loss affects urogenital tissues adversely [13,14,15].

Despite the prevalence, multiple studies have shown that the majority of patients with pelvic floor disorders (PFDs), especially elderly women, do not discuss their problem with a physician, even though the incontinence is severe. The severity of the problem correlates with treatment seeking [16,17].

Multiple barriers to care seeking for urinary incontinence have been identified, including embarrassment and shame, the misperception that urinary incontinence is a normal part of aging, the view that UI was less important compared to other medical problems, and lack of knowledge of effective treatment options [18].

The view that UI is a natural part of aging is still alive and well, and few elderly women receive proper treatment. Older patients are reluctant to undergo surgery. Drugs used for the treatment of UI often cause unacceptable side effects in the elderly and it is difficult to learn and maintain a program of pelvic floor exercises [10].

On the other hand, a wide range of treatment options is available. First-line management includes lifestyle and behavioral modification, pelvic floor exercises, and bladder training.

Anticholinergic drugs and mirabegron (a beta-3 agonist) are the basic pharmacological treatment of urge UI (UUI). Anticholinergics are used as a second-line treatment for UUI and bladder overactivity. Discontinuation because of adverse effects such as dry mouth, constipation, blurred vision, or fatigue is frequent. A recent longitudinal cohort study showed a risk of deterioration in cognitive function and brain atrophy with prolonged use of anticholinergics, particularly in the elderly [19].

Mirabegron is also available and its cardiovascular safety is comparable with that of anticholinergics. The most common adverse events are hypertension, nasopharyngitis, and urinary tract infection [20].

Estrogens and other pharmacological interventions are helpful in the treatment of urgent incontinence that does not respond to conservative measures. Estrogen treatment might be initiated earlier as a preventive medicine and better diagnosis of the type of incontinence before writing the prescription may yield better results. Estrogens have been used to treat incontinence in postmenopausal women for many years, either alone or in combination with other drugs (such as anticholinergic agents), and there is evidence that urinary incontinence may improve with local estrogen treatment. There are insufficient high-quality data to support the use of vaginal estrogens for SUI after menopause. The evidence regarding local estrogens and OAB in postmenopausal women is credible. Local estrogen therapy improves voiding function and decreases the risk of developing OAB syndrome. Vaginal estrogens cause changes in autonomic and sensory vaginal innervation and may decrease urothelial damage, inflammatory cell infiltrations, and muscular atrophy [10,20].

Third-line therapies (e.g., sacral neuromodulation, intravesical onabotulinum toxin-A injections, and posterior tibial nerve stimulation) are useful in selected patients with refractory urge incontinence.

Onabotulinum toxin A has been licensed in Europe to treat OAB with persistent or refractory UUI since 2011. The mechanism of action is to block the presynaptic release of acetylcholine, thereby reducing the activation of muscarinic receptors and detrusor muscle contraction. The most important adverse events are urinary tract infections and an increase in post-void urinary residue that may require temporary intermittent catheterization [20].

Surgery should be considered in postmenopausal women with stress urinary incontinence (SUI). Midurethral slings, including retropubic and transobturator approaches, are safe and should be offered. In our study group, surgical treatment of stress urinary incontinence was performed in 123 patients. The most commonly used technique (*n* = 115) in the treatment of SUI was transobturator tape (TOT) placement. Only one case of an intraoperative complication was noted—bladder perforation, which was simultaneously repaired. There were no serious postoperative complications reported.

In a prospective trial comparing women >80 years old and <80 years old undergoing midurethral sling procedures for SUI, the authors showed no difference in overall cure rate between both groups. Hospital length of stay was significantly longer for the older group. Major perioperative complications were uncommon and the rates of bladder perforation, long-term voiding difficulty, and de novo urgency UI were similar between the groups [21].

Serati et al. evaluated the efficacy and safety of the transobturator approach for the surgical management of SUI in two groups: >70 years old and <70 years old. The main conclusion was that there are no significant differences between the two groups in terms of cure rate, voiding dysfunction, vaginal exposition and persistent groin pain, or onset of the novo overactive bladder [22].

Ellington et al. reviewed outcomes of surgery for SUI among older women and found that functional and cognitive impairment in the aging population and age-related changes in lower urinary tract (LUT) function were complicating factors in the surgical treatment of SUI. In fact, LUT symptoms in octogenarians should be considered as a component of a geriatric syndrome [23].

Urethral bulking agents should not be offered as first-line treatment, particularly in patients desiring a long-term solution for SUI, but could be considered in specific postmenopausal populations such as women with recurrent or persistent SUI or in the elderly, for whom a minimally invasive approach is preferred [20]. Urethral bulking agents have been documented to improve SUI symptoms in women who either do not want to undergo more invasive surgery or who are not surgical candidates because of medical comorbidity. They are divided into two categories: collagen (which degrades over a 6-to-12-month period of time) and nondegradable synthetic agents. The advantages of injectable urethral bulking agents, especially in older women, include the fact that it is a procedure easily performed in an office setting, that many women tolerate it well without anesthesia, and that anticoagulation does not always need to be stopped before injections. Complications include urinary retention and urinary tract infection, allergic reaction to collagen, and bead migration of nondegradable synthetic agents with rare events, such as distant arterial thrombosis reported, vaginal and urethral erosion, and periurethral abscess [23].

Laser treatment was developed to induce neocollagenogenesis to increase the thickness and strength of the anterior vaginal wall for better support of the bladder and urethra and thus an improvement in continence. Although the data for SUI treatment were encouraging, several issues could limit the use of that technology. The effectiveness of this modality for severe SUI is very low for older women and those who are overweight or obese [12].

The relative risk of complications in older women, especially those aged over 80, was significantly increased compared with younger women. Ellington et al. draw attention to the fact that many institutions have reported excellent surgical results with well-selected octogenarians and nonagenarians undergoing surgeries for incontinence and other pelvic floor disorders, but these findings describe healthy, well-selected older women hospitalized at specialized centers [23].

A few studies mainly focus on midurethral slings and reveal similar efficacy and safety profiles for octogenarian women in comparison to younger women. The data suggest that greater frailty, not older age, may be associated with complications in the elderly population [24,25,26].

As life expectancy worldwide continues to increase, it is increasingly common to see patients in their 80 s presenting with UI. This age group is prone to multiple comorbidities, functional disability, and/or cognitive impairments [24]. Complications did not increase with age, although short-term voiding difficulties were more frequent in the older age group [27].

It is estimated that between 11 and 19% of females undergo surgery for prolapse or incontinence by the age of 80 to 85 years, with 30% of these individuals potentially requiring additional prolapse repair procedures. The choice of the primary surgical approach for patients with POP is contingent upon various factors, including overall health status and the patient’s preferences [28].

The research indicates a notable rise in complications associated with prolapse surgery in patients older than 80, irrespective of frailty and other risk factors. Despite these challenges, the vaginal approach has been identified as the safest surgical method for pelvic organ prolapse (POP) repair in the elderly population.

The approximate rate of severe complications stands at around 9 to 25% among the very elderly population [28].

Other studies have shown that women aged over 80 years undergoing surgery for POP have a 13.6 higher risk of postoperative death than their counterparts. However, it was not the case in our study group.

The main concerns while treating geriatric patients are the following: functional aging with myogenic and neurological changes, polypharmacy and impaired cognitive function, and risk of delirium [29,30].

The patients in this age group also have higher risks of cardiac complication, stroke, and mortality. Certain specific complications, including the need for blood transfusion or the experiencing of significant blood loss, as well as occurrences like pulmonary edema and postoperative congestive heart failure, have been found to be correlated with various risk factors. These risk factors encompass aspects such as the duration of surgery, the presence of coronary artery disease, and the existence of peripheral vascular disease [28].

The prevalence of acute postoperative urinary retention (POUR) subsequent to pelvic reconstructive surgery, impacting approximately 15–45% of women, does not appear to exhibit any correlation with frailty among older women undergoing prolapse surgery. This suggests that the occurrence of POUR in this context is not influenced by the frailty status of older female patients undergoing prolapse surgery [31]. In our study, postoperative urinary retention occurred in only 11 patients out of 245 pelvic floor reconstruction surgeries, accounting for 4.49%.

The vaginal approach has been demonstrated to be the safest overall surgical approach for POP repair in the elderly population. It usually takes a shorter operating time and can be performed even under regional anesthesia. Currently, there is a renewed interest among scholars in native tissue techniques for pelvic organ prolapse repair, primarily driven by considerations of cost-effectiveness and the avoidance of complications associated with mesh application [28,32]. Current recommendations suggest that vaginal mesh repair should only be offered in high-risk individuals where the benefit of mesh placement can justify the risk of such procedures [33].

Therefore, vaginal meshes in our department were offered only to patients with vaginal vault prolapse when we used a well-standardized technique that addressed both apical and anterior defects [34].

Obliterative vaginal procedures are a good option for elderly patients who cannot tolerate extensive surgery and who are not planning future sexual activity. The geriatric patients who underwent either reconstructive or obliterative procedures were relieved of their preoperative symptoms and their quality of life had greatly improved [32].

In the follow-up study presented by Kissane, the authors showed that older women (age ≥ 70) not only achieved similar surgical success compared to younger women undergoing primary transvaginal apical prolapse repair, but also showed a greater improvement in pelvic floor distress symptoms, specifically prolapse bother symptoms. Complication rates, length of stay, and retreatment rates were similar between groups, and both older and younger women showed improvement in the PFDI-20 questionnaire assessment [35].

To achieve the optimal management outcomes in surgical geriatric and high-risk group patients, it is vital to have the dedicated collaboration and coordination of primary care, geriatric medicine, social service, and other allied health services to adequately assess physical status, cognition, and frailty. A patient-centered multimodal and multidisciplinary model that includes a focused pathway of perioperative and postoperative management should be further investigated.

Therefore, it is presented as an integral part of the ERAS (accelerated recovery after surgery) program aimed at building physiological reserve, especially in the geriatric high-risk group, and better adaptation to surgical stress [36].

Older women undergoing pelvic organ prolapse repair not only derive meaningful benefits from surgery but also experience a low rate of complications. Körnig et al. stated that chronological age alone should not be regarded as a contraindication to operative treatment. Clinical decision-making should instead be individualized, and further research in this expanding population is needed to better assess the safety and effectiveness of surgical interventions [37].

Although safety outcomes in our research appeared favorable, there are some limitations that should be highlighted. Our study’s retrospective character limits the ability to determine causality and frailty—known to strongly influence perioperative outcomes. Additionally, long-term postoperative outcomes, recurrence rates, functional improvement, and quality-of-life effects were not assessed. This study lacks a control group, which prevents direct comparison with younger cohorts. The limitation significantly restricts the ability to determine relative risk. Therefore, all conclusions must be confined only to the study group.

Despite these limitations, the study adds valuable evidence regarding short-term surgical safety in elderly women.

## 5. Conclusions

The growing proportion of elderly women, particularly those over 80 years of age, presents a significant and evolving challenge for modern urogynecology.

In this 10-year retrospective cohort of women aged ≥ 80 years, urogynecological surgery demonstrated a low rate of major perioperative complications. These findings suggest that, despite advanced age and the frequent presence of comorbidities and polypharmacy, surgical management of pelvic floor disorders in this population is feasible.

In our analysis, pelvic organ prolapse, stress urinary incontinence, and overactive bladder were the most common indications for urogynecological intervention. The majority of patients qualified for surgical treatment, with vaginal native tissue repair being the predominant approach. Obliterative procedures such as LeFort colpocleisis were appropriately offered to non-sexually-active women.

The overall rate of complications was low, and postoperative urinary retention occurred in only 4.49% of cases, with no major long-term consequences observed. Importantly, chronological age alone should not be considered a contraindication to surgery. Instead, frailty assessment and individualized risk stratification are essential to guide clinical decision-making.

Future prospective studies are needed to further clarify long-term outcomes and refine surgical selection criteria for this population.

## Figures and Tables

**Table 1:** Parity of the analyzed group.

Nulliparous	N = 24		
Only cesarean section	N = 4		
Vaginal delivery and cesarean section	N = 11	1 Vaginal Delivery	N = 42
		2 Vaginal Deliveries	N = 203
Only vaginal delivery	N = 430		
		≥3 Vaginal Deliveries	N = 196

**Table 2:** The most common comorbidities of the patients.

Hypertension	84.22% (N = 395)
Diabetes mellitus type 2	21.1% (N = 99)
Hypothyroidism	12.15% (N = 57)

**Table 3:** Charlson Comorbidity Index (CCI) assessment of the group.

Number of Patients (%)	Charlson Comorbidity Index Results (Points, % Chance of 10-Years Survival)
N = 312 (66.52%)	4 (53%)
N = 132 (28.14%)	5 (21%)
N = 21 (4.48%)	6 (2%)
N = 3 (0.85%)	7 (0%)
N = 1 (0.21%)	8 (0%)

**Table 4:** POP-Q scale assessment among patients with prolapse.

POP-Q Scale	Number of Patients (*n*; %)
II	N = 13 (5%)
III	N = 120 (50%)
IV	N = 109 (45%)

**Table 5:** The complications according to Clavien–Dindo scale.

Clavien–Dindo Classification	Number of Patients	Type of Treatment Required
Grade 1	N = 43	Additional pharmacotheraphy, e.x. antiemetics, anti-pyretics or opioid painkillers
Grade 2	N = 12	N = 1—red blood cell concentrate transfusion N = 11—acethylcholinesterase inhibitors and clean intermittent catheterization due to urine retention
Grade 3a	N = 1	Evacuation of the hematoma of posterior vaginal wall in local anesthesia

**Table 6:** The complications according to Clavien–Dindo scale among patients suffering from SUI.

Clavien–Dindo Classification	Number of Patients	Type of Treatment Required
Grade 1	N = 19	Additional pharmacotheraphy, e.x. antiemetics and anti-pyretics.
Grade 2	N = 6	Acethylcholinesterase inhibitors and clean intermittent catheterization due to urine retention
Grade 3b	N = 1	Removal of the tape in general anesthesia due to urine retention

## Data Availability

The raw data supporting the conclusion of this article will be made available by the corresponding author on request.

## Abbreviations

LUTS	Lower Urinary Tract Symptoms
FDA	Food and Drug Administration
CCI	Charlson Comorbidity Index
POP	Pelvic Organ Prolapse
VNTR	Vaginal Native Tissue Repair
TVH	Transvaginal Hysterectomy
TVM	Transvaginal Mesh Insertion
SUI	Stress Urinary Incontinence
TOT	Transobturator Midurethral Sling
TVT	Tension-free Vaginal Tape
OAB	Overactive Bladder
MUI	Mixed Urinary Incontinence
VVF	Vesicovaginal Fistula
UI	Urinary Incontinence
IUP	Intraurethral Pressure
UTI	Urinary Tract Infection
PFD	Pelvic Floor Disorders
UUI	Urge Urinary Incontinence
POUR	Postoperative Urinary Retention
PFDI-20	Pelvic Floor Distress Inventory-20
ERAS	Enhanced Recovery After Surgery
